# Leishmaniasis: A spectrum of diseases shaped by evolutionary pressures across diverse life cycle

**DOI:** 10.1093/emph/eoaa032

**Published:** 2020-07-27

**Authors:** Alejandro L Antonia, Dennis C Ko

**Affiliations:** e1 Department of Molecular Genetics and Microbiology, School of Medicine, Duke University, Durham, NC 27710, USA; e2 Division of Infectious Diseases, Department of Medicine, School of Medicine, Duke University, Durham, NC 27710, USA

## LEISHMANIASIS

Leishmaniasis is a vector-born disease, caused by *Leishmania* genus protozoan parasites*.* Inoculation by infected *Phlebotamine* sandflies results in asymptomatic infection or a diverse range of clinical manifestations. Leishmaniasis can present with tegumentary lesions or visceral disease with parasite dissemination and high mortality [1]. The spectrum of tegumentary disease includes simple-cutaneous, diffuse, disseminated and mucocutaneous lesions. Around 20 of more than 50 described *Leishmania* species cause this spectrum of human disease [2]. However, the link between parasite diversity and disease outcome remains incompletely understood because of host and environmental modifiers influencing evolutionary fitness.

## EVOLUTIONARY PERSPECTIVES

**Figure 1.**
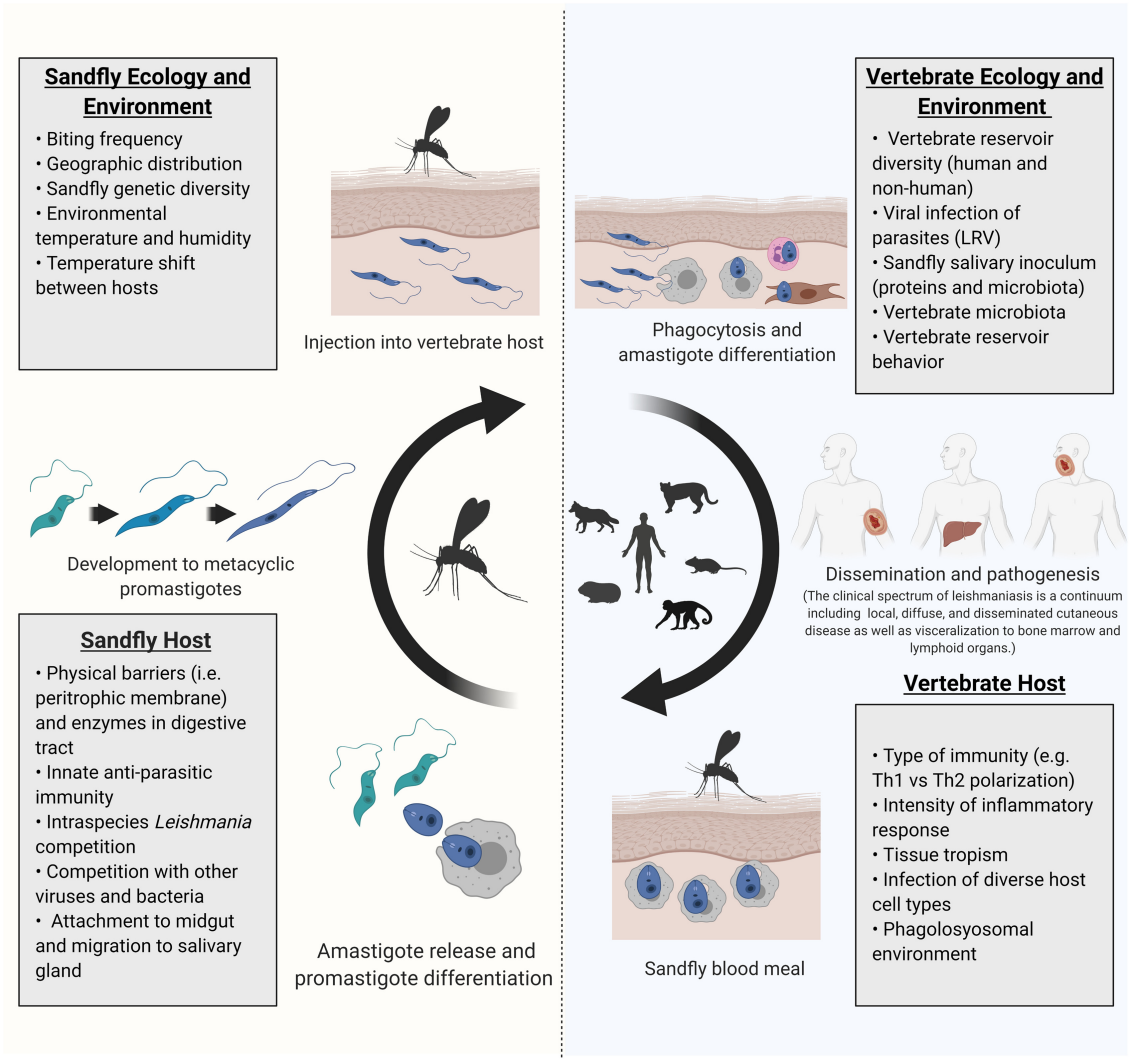
Diverse evolutionary pressures across Leishmania life cycle

Evolutionary biology can be applied to understand the genetic differences among parasites with different disease manifestations. Because *Leishmania* parasites encounter diverse environments, genetic adaptations may result in positive or negative trade-offs in other life-cycle stages. In addition, as humans are not the primary vertebrate reservoir, protective or pathologic immune responses may alter disease outcome but not parasite fitness. Thus, understanding how *Leishmania* genetics influences outcomes of human leishmaniasis requires consideration of selective pressures across life-cycle stages (Fig. 1). For instance, fitness in the sandfly is influenced by immunologic barriers, microbial competition and insect ecology [2, 3]. In vertebrate hosts, fitness is influenced by the immune response, tissue tropism and vertebrate ecology [4]. Leishmaniasis persists in part due to genetic diversity and genomic plasticity [5] allowing *Leishmania* parasites to navigate its evolutionary landscape.

## FUTURE IMPLICATIONS

While the complex pressures shaping *Leishmania* evolution provide challenges to investigating host range and parasite virulence, they also present opportunities to understand human susceptibility. The range of leishmaniasis is limited by interactions between vectors and animal reservoirs. As their habitat is altered due to climate change and social conflict, ecologic studies informed by evolutionary dynamics could predict changes in leishmaniasis distribution. Human pathogenesis varies with the infecting *Leishmania* species; however, infection with the same species can result in an appropriate immune response with resolution, impaired response with parasite proliferation, or excessive inflammation with immunopathology [4]. To elucidate human contributions to this variation, epidemiologic and genome wide association studies are needed that account for parasite diversity. Furthermore, as *Leishmania* parasites encode mechanisms to influence host immunity, understanding molecular pathogenesis requires studies using a diverse collection of parasites. Synthesis of these approaches is needed to understand variation in leishmaniasis outcomes and improve human health.

This evolutionary complexity may prove advantageous in developing novel treatments. For example, targeting parasites in vectors and reservoirs can decrease transmission and minimize human toxicity. This strategy has shown promise against *Plasmodium* parasites by treating mosquito nets with anti-malarial compounds [6]. Furthermore, host-directed therapies can be designed with decreased risk of drug resistance by exploiting pathways that are anti-parasitic in the incidental human host but not the primary animal reservoir. This represents a novel strategy for ‘evolution-proof’ therapies [7] uniquely available for zoonotic pathogens.
